# A novel fluorescent biosensor based on dendritic DNA nanostructure in combination with ligase reaction for ultrasensitive detection of DNA methylation

**DOI:** 10.1186/s12951-019-0552-5

**Published:** 2019-12-07

**Authors:** Shu Zhang, Jian Huang, Jingrun Lu, Min Liu, Yan Li, Lichao Fang, Hui Huang, Jianjun Huang, Fei Mo, Junsong Zheng

**Affiliations:** 1 Department of Clinical and Military Laboratory Medicine, College of Medical Laboratory Science, Army Medical University, Chongqing, 400038 China; 2 Department of Basic Clinical Laboratory Medicine, School of Clinical Laboratory Science, Guizhou Medical University, Guiyang, 550004 China; 3 Center for Clinical Laboratories, Affiliated Hospital of Guizhou Medical University, Guiyang, 550004 China; 4 Department of Breast Surgery, Affiliated Hospital of Guizhou Medical University, Guiyang, 550004 China

**Keywords:** Fluorescent biosensor, DNA methylation, Dendritic DNA, Catalyzed hairpin assembly, Ligation detection reaction

## Abstract

**Background:**

DNA methylation detection is indispensable for the diagnosis and prognosis of various diseases including malignancies. Hence, it is crucial to develop a simple, sensitive, and specific detection strategy.

**Methods:**

A novel fluorescent biosensor was developed based on a simple dual signal amplification strategy using functional dendritic DNA nanostructure and signal-enriching polystyrene microbeads in combination with ligase detection reaction (LDR). Dendritic DNA self-assembled from Y-DNA and X-DNA through enzyme-free DNA catalysis of a hairpin structure, which was prevented from unwinding at high temperature by adding psoralen. Then dendritic DNA polymer labeled with fluorescent dye Cy5 was ligated with reporter probe into a conjugate. Avidin-labeled polystyrene microbeads were specifically bound to biotin-labeled capture probe, and hybridized with target sequence and dendritic DNA. LDR was triggered by adding Taq ligase. When methylated cytosine existed, the capture probe and reporter probe labeled with fluorescent dye perfectly matched the target sequence, forming a stable duplex to generate a fluorescence signal. However, after bisulfite treatment, unmethylated cytosine was converted into uracil, resulting in a single base mismatch. No fluorescence signal was detected due to the absence of duplex.

**Results:**

The obtained dendritic DNA polymer had a large volume. This method was time-saving and low-cost. Under the optimal experimental conditions using avidin-labeled polystyrene microbeads, the fluorescence signal was amplified more obviously, and DNA methylation was quantified ultrasensitively and selectively. The detection range of this sensor was 10^−15^ to 10^−7^ M, and the limit of detection reached as low as 0.4 fM. The constructed biosensor was also successfully used to analyze actual samples.

**Conclusion:**

This strategy has ultrasensitivity and high specificity for DNA methylation quantification, without requiring complex processes such as PCR and enzymatic digestion, which is thus of great value in tumor diagnosis and biomedical research.

## Background

DNA methylation is one of the earliest and most well-researched approaches for epigenetic modification, with the main process of transferring a methyl group to a cytosine residue at the 5′ end of CpG dinucleotide (5′-CG-3′) catalyzed by methyltransferase using S-adenosylmethionine as a donor [1–4]. Abnormal DNA methylation may lead to imbalances of cell functions including DNA replication and repair, gene transcription, X-chromosome inactivation, genomic imprinting and cell differentiation, which is closely related to the onset and progression of many cancers and thus commonly used as the biomarker to assess cancer risk and prognosis [5–9]. For instance, the tumor suppressor genes of breast cancer are inactivated by abnormal methylation of the promoter region. When the CpG island of the promoter region is hypermethylated, the expression of tumor suppressor gene may be decreased or even suppressed, thus inducing cancer onset, progression and even metastasis [10–13]. Accordingly, detecting the DNA methylation level has become a new strategy for the clinical diagnosis, early warning and susceptibility analysis of cancers.

In the past few decades, researchers have endeavored to develop feasible analytic methods for DNA methylation. Currently, methylation is detected mainly depending on three methods, i.e. methylation sensitive restriction endonuclease analysis established by restriction endonuclease-specific cleavage [14–17], genomic methylation sequencing established on the basis of bisulfite conversion [18–22] and methylation array analysis [23–26]. However, all the above methods have limitations, such as expensive apparatus, complicated operation, low sensitivity, and narrow detection range which is susceptible to recognition by restriction endonuclease, so their clinical applications are limited. The above issues have been partly solved by recently emerging technologies. For example, Wang et al. detected DNA MTase activity based on single-ribonucleotide repair-mediated ligation-dependent cycling signal amplification, with the limit of detection (LOD) of as low as 4.8 × 10^−6^ U/mL [27]. Based on a dual signal amplification strategy combining terminal deoxynucleotidyl transferase (TdT)-assisted enzymatic amplification with Ru(III) redox cycling, Cui et al. detected DNA methylation by using a label- and immobilization-free electrochemical magnetobiosensor. Cytosine, 5-methylcytosine and 5-hydroxymethylcytosine, as three major epigenetic variants in DNA bases, were then accurately quantified and distinguished [28]. Besides, Huang et al. quantified multiple DNA methylation sites through specific capture of methylated cytosine by graphene-conjugated anti-5-methylcytosine antibody. The resulting steric hindrance effect was used to localize a single methylation site [29]. In addition, Wu et al. selectively labeled 5-hydroxymethylcytosine in aqueous solution using various sulfonate reagents under non-enzymatic reaction conditions. This method allowed an efficient single binding step of biotin to hydroxymethylcytosine in DNA for accurate identification [30]. Moreover, Yotani et al. [31] developed an anion column to accurately distinguish methylated and unmethylated DNAs by high-performance liquid chromatography based on electrostatic and hydrophobic properties.

Fluorescent biosensors combine specific molecular recognition events with fluorescence conversion processes [32, 33]. Fluorescence sensing systems have the advantages of facile operation, real-time detection, high throughput and easy automation [34–36], and have overcome the limitations of the above methods and thus attracted widespread attention [37–40]. Wang et al. developed a label-free fluorescence method through ligation-mediated rolling-circle amplification based on specific oxidation of 5-hydroxymethylcytosine to 5-formylcytosine and then conversion into uracil. As a result, 5-hydroxymethylcytosine and 5-methylcytosine were well distinguished [41]. Subsequently, their group developed a fluorescence-based method to sensitively detect DNA adenine methyltransferase on the basis of TdT-activated endonuclease IV-assisted hyperbranched amplification [42]. Nevertheless, detecting DNA methylation with fluorescence biosensors is still limited, especially owing to false negativity caused by low analyte concentration and sensitivity in actual sample detection. Therefore, it is necessary to design a signal amplification strategy to augment the sensitivity of a fluorescent biosensor [43]. Dendritic DNA polymers are often used as the signal amplification tags for biosensors because of excellent performance, biostability and biocompatibility [44, 45]. Dendritic DNA nanostructures are conventionally constructed based on Watson–Crick hydrogen bond interaction. Briefly, three single strands are designed and annealed to synthesize sticky Y-DNA which is further hybridized with other Y-DNAs to gradually construct dendritic DNA. However, this traditional method is both time-consuming and laborious, also requiring high-concentration DNA [46, 47]. Thus, an efficient and low-cost dendritic DNA nanostructure is in great demand to elevate the sensitivity of a fluorescent biosensor.

Since methylated and non-methylated DNA sequences differ only at a single site after bisulfite treatment, an eligible method should have sufficiently high specificity. This requirement can hardly be met by classical oligonucleotide arrays, and the results may be false-positive [48]. Ligation detection reaction (LDR), which can distinguish the specificity of single nucleotide mutation [49], has become one of the most promising analytic methods for methylation and other epigenetic modifications. LDR requires two oligonucleotide probes for each target sequence. Only in the case of a perfect match, the two probes can form a duplex with the target sequence, so base mismatch sequences can be recognized selectively [50].

**Fig. 1:**
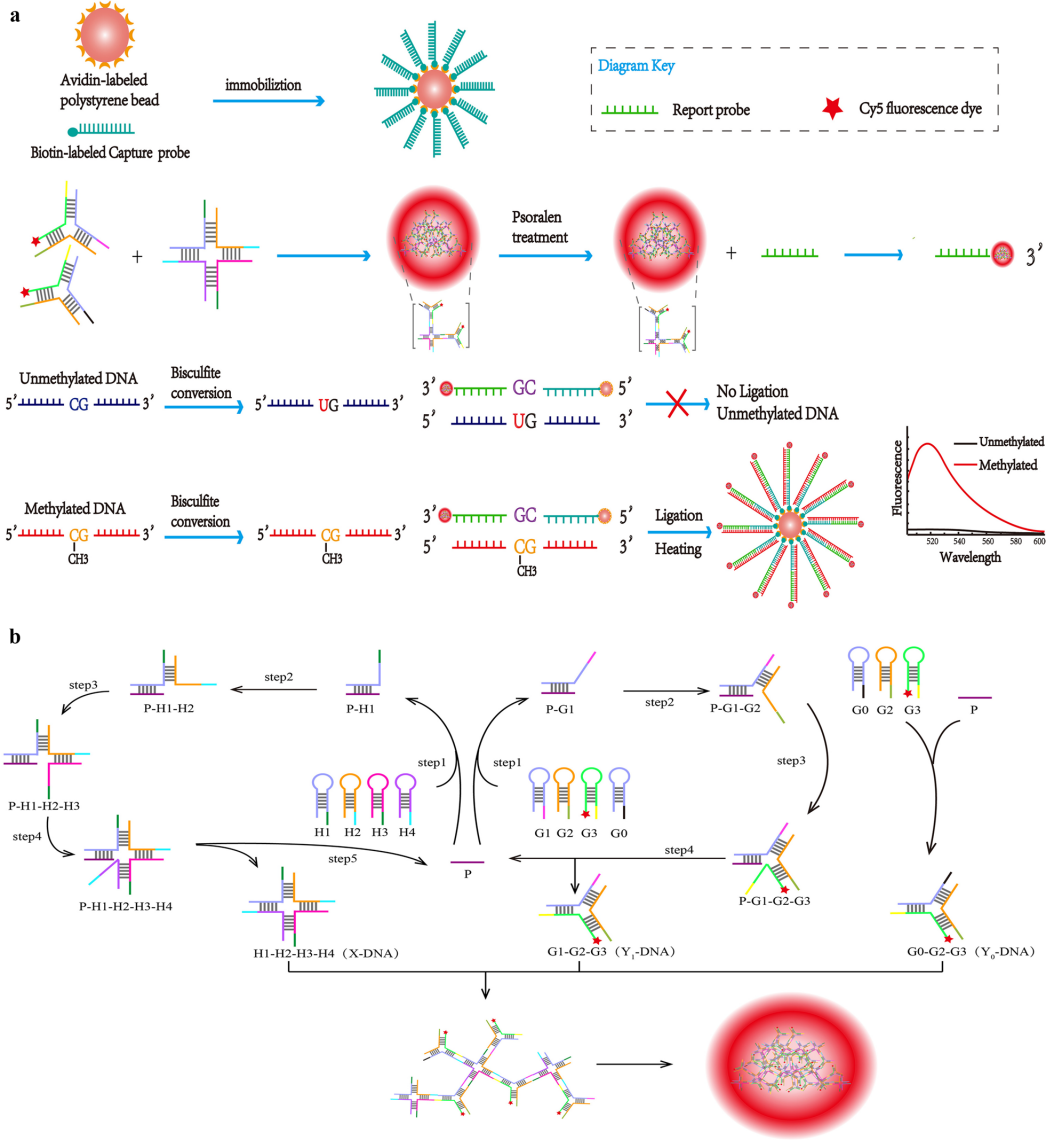
Detailed process of the proposed method. **a** Scheme of DNA methylation detection by using the developed fluorescence biosensor. **b** Scheme for formation of dendritic DNA polymer. Identical DNAs are marked by the same color

To further augment the sensitivity and specificity of a fluorescent biosensor for DNA methylation, we herein developed a fluorescence detection strategy which used dendritic DNA polymer formed by enzyme-free DNA-catalyzed hairpin assembly for signal amplification in combination with LDR, without needing PCR amplification or restriction enzymatic digestion. The polymer was fabricated by employing Y-DNA and X-DNA as the donor and core acceptor molecule, respectively, and self-assembling Y-DNA into X-DNA along a designated direction. This strategy gave a larger polymer than the conventional one prepared by assembling Y-DNA alone to reach saturation at a certain generation. As a result, the detection signal was obviously amplified, and the time of preparing dendritic DNA through enzymatic reaction can be shortened when the enzyme-free method was utilized. In the meantime, the detection specificity was substantially raised by combining with LDR. The detailed process is schematized in Fig. 1a. Firstly, the promoter fragment of human breast cancer BRCA1 gene was selected as the target sequence and treated with bisulfite to generate a DNA sequence difference between methylated and unmethylated cytosines. Secondly, the key elements of dendritic DNA, i.e. X-DNA and Y-DNA, were synthesized by the Toehold-induced strand displacement reaction catalyzing the hairpin structure, and then gradually self-assembled. Afterwards, psoralen was added to prevent DNA from unwinding at high temperature, and dendritic DNA polymer labeled by fluorescent dye Cy5 was ligated with reporter probe into a conjugate. Subsequently, avidin-labeled polystyrene microbeads were specifically bound to biotin-labeled capture probe and hybridized with the target sequence and dendritic DNA. LDR was thereafter triggered by adding Taq ligase. When cytosine was methylated, the capture probe and reporter probe completely matched the target sequence and formed a stable duplex. In contrast, unmethylated cytosine that underwent bisulfite treatment was converted into uracil to cause a base mismatch instead of forming a duplex. The methylation status of target sequence fragment can thus be identified by analyzing its fluorescence signal. Meanwhile, this method was successfully applied to detect the methylation status of BRCA1 promoter in actual samples, suggesting potential values in early cancer diagnosis and treatment outcome evaluation.

## Results and discussion

### Mechanism for synthesis of dendritic DNA polymer

The DNA hairpin structures designed in this study had partially complementary sequences with overhanging sticky ends. Since most complementary sequences were in the stem loop of hairpin, the designed hairpin structures cannot spontaneously hybridize, so the original ones were maintained when there was no excitation probe. After excitation probe was added, the hairpin structure of H1 was opened, producing a P-H1 intermediate. Then the newly exposed fragment of H1 was bound to the sticky end of H2, which triggered the second strand displacement reaction, forming a P-H1-H2 complex. Finally, the hairpin structures of H3 and H4 were opened in the same way, which generated an unstable P-H1-H2-H3-H4 complex, readily forming X-DNA after the excitation probe was displaced by the exposed fragment of H4. The displaced excitation probe continued to trigger another hairpin probe to produce new X-DNA. Y-DNA was gradually constructed in the same way.

To fabricate dendritic DNA, Y-DNA and X-DNA were employed as the donor and core acceptor molecule, respectively. Its orientation was controlled by the sticky end sequences of each branch of X-DNA, and Y-DNA was spontaneously assembled into the receptor X-DNA along a specific direction. In detail, during the formation of dendritic DNA, one arm of Y0-DNA was occupied by fluorescent dye, one arm was designed to complement the reporter probe, and the remaining one was bound to X-DNA. The remaining three arms of X-DNA bound two arms of Y1-DNA, followed by similar processes. A dendritic DNA polymer was eventually obtained (Fig. 1b). During preparation, only 100 nM DNA was required, which was 400-fold lower than those of conventional methods [46, 47]. Besides, the preparation time was shortened from 2–5 days to 2 h. Therefore, the disadvantages of high DNA concentration and long synthesis time of dendritic DNA can be circumvented. Particularly, this method gave a larger polymer than the conventional one prepared by assembling Y-DNA alone to reach saturation at a certain generation. Additionally, each Y-DNA molecule emitted fluorescence signal, thereby facilitating signal capture and markedly elevating the biosensor sensitivity. This dendritic DNA had a three-dimensional structure with high density, which prevented against nuclease degradation, thus being beneficial to application in real samples as confirmed below.

### Characterizations of X-DNA, Y-DNA, dendritic DNA polymer and avidin-coated microbeads

**Fig. 2:**
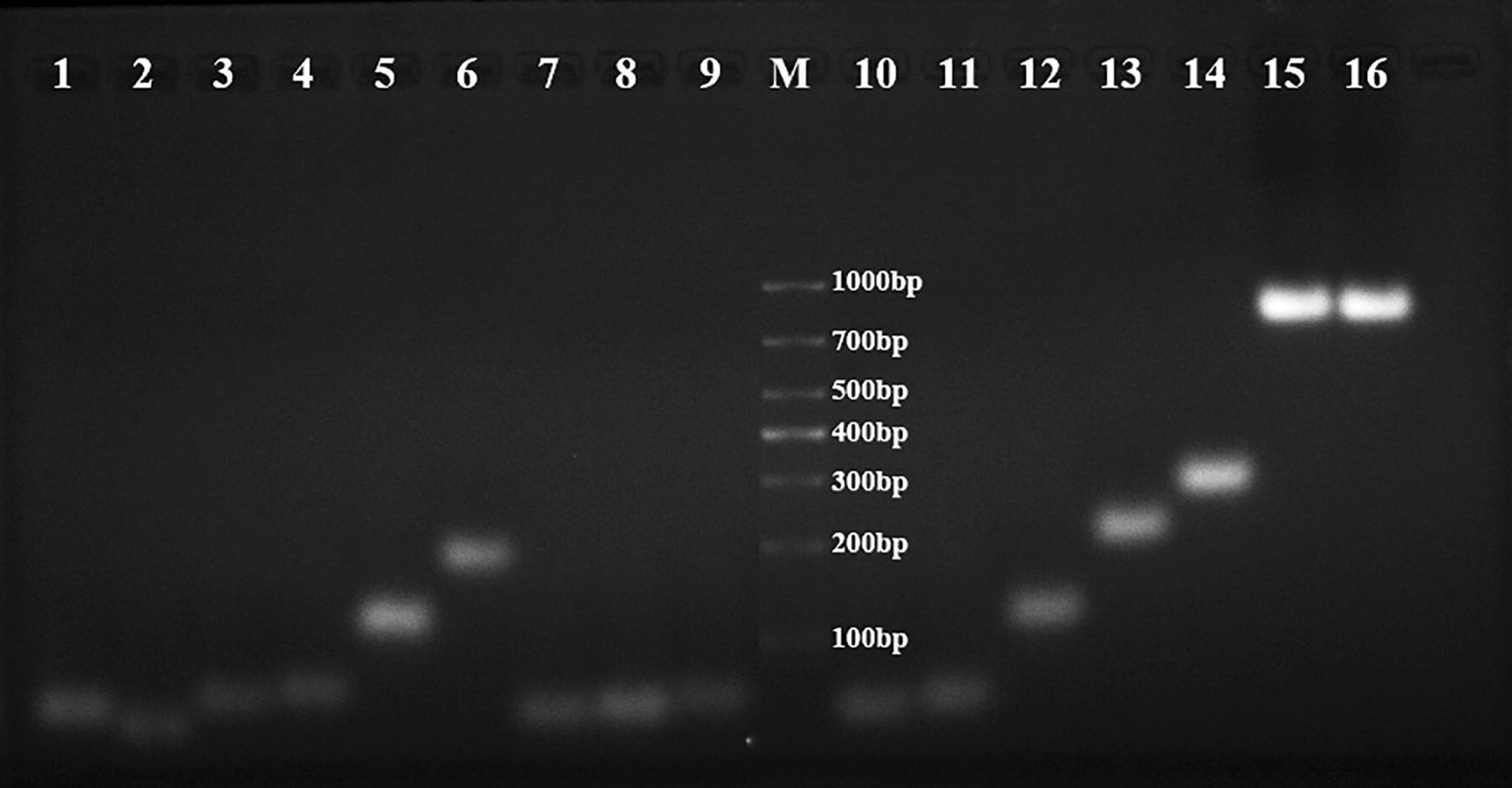
Agarose gel electrophoresis results of different DNA structures. Lanes 1–3: Hairpin structures G1, G2 and G3; lane 4: intermediate P1-G1; lane 5: intermediate P1-G1-G2; lane 6: Y-DNA; lanes 7–10: hairpin structures H1, H2, H3 and H4; lane 11: intermediate P1-H1; lane 12: intermediate P1-H1-H2; lane 13: intermediate P1-H1-H2-H3; lane 14: X-DNA; lane 15: dendritic DNA; lane 16: dendritic DNA after 60 days of storage at 4 °C; M: DNA marker

X-DNA and Y-DNA were constructed as scaffolds for dendritic DNA, which was evaluated by agarose gel electrophoresis (Fig. 2). The rate of DNA migration depends on base number and shape as well as degree of base pairing [51]. The bands of hairpin structures G1, G2, G3, H1, H2, H3, H4 and intermediates P-H1, P-G1 (lanes 1–4, lanes 7–11) migrated fastest. An obvious band appeared in the right lane (lane 5, lane 12), with the migration being slightly lower than that of intermediates. Since P-G1-G2 and P-H1-H2 complexes indeed formed, two arms of DNA had been successfully constructed. The migration of P-G1-G2-G3 and P-H1-H2-H3-H4 products (lane 6, lane 14) was as expected, which demonstrated successful assembly of Y-DNA and X-DNA. The band of dendritic DNA (lane 15) migrated slowest. There were no obvious by-products, so the yield and purity were both high. Given that the band of the dendritic DNA sample stored at 4 °C for 60 days (lane 16) remained almost unchanged, it was highly stable.

Atomic force microscopy (AFM) (Additional file 1: Figure S1A), transmission electron microscopy (TEM) (Additional file 1: Figure S1B) and dynamic light scattering (DLS) (Additional file 1: Figure S1C) results revealed that the prepared DNA nanopolymer had consistent structure and size with those reported previously [52, 53], but the diameter was as large as (91.2 ± 6.7) nm. Meanwhile, scanning electron microscopy (SEM) showed that both unmethylated LDR product (UMMB, Additional file 1: Figure S2A) and methylated LDR product (MMB, Figure S2B) were spherically shaped, with the average particle size of 5.5 μm. MMB had a rougher surface than that of UMMB due to covering with the dendritic structure.

### Feasibility of sensing system

Non-denaturing gel electrophoresis was conducted to analyze methylated and unmethylated LDR products. The band of ligation product has a consistent length with that of the target. As shown in Additional file 1: Figure S3, the 50 nt bands in lanes 1 and 2 represent methylated and unmethylated target DNAs, respectively. Lanes 3 and 4 correspond to the bands of unligated capture probe (35 nt) and reporter probe (33 nt), respectively, and the band at 68 nt in lane 5 represents the ligated product of capture probe and reporter probe. In the case of unmethylated DNA, three bands at about 700 nt, 50 nt and 35 nt are present (lane 6), suggesting that a single mismatch of guanine–uracil cannot induce LDR. In the presence of methylated DNA, a band at approximately 785 nt (lane 7) appears, indicating that methylated DNA successfully induced DNA-probe ligation.

**Fig. 3:**
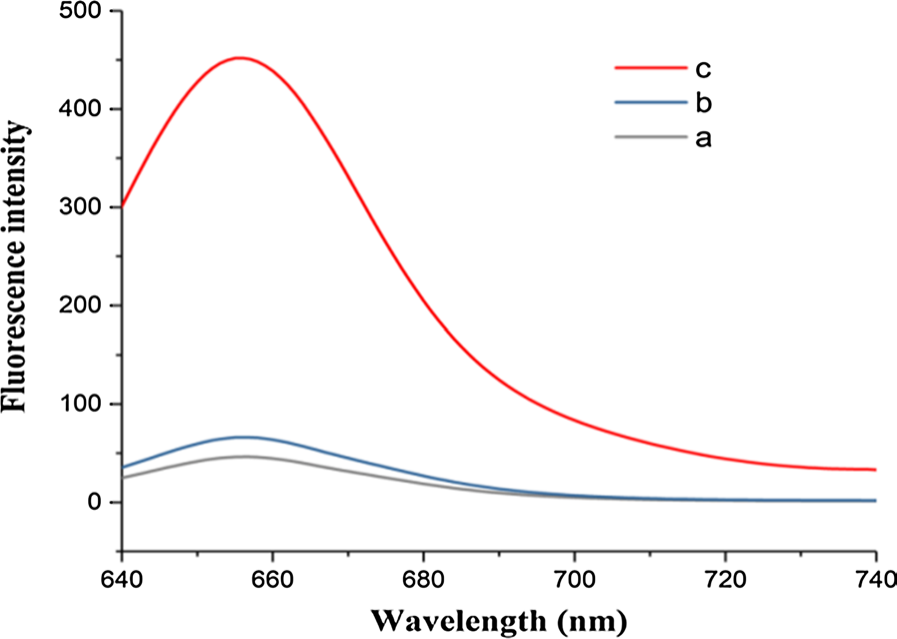
Fluorescence emission spectra of methylated (red) and unmethylated (blue) DNAs. Curve *a*: Blank control; curve *b*: unmethylated sample; curve *c*: methylated sample. Concentrations of methylated and unmethylated DNAs: 1 pM

Subsequently, the feasibility of the constructed fluorescent biosensor for DNA methylation detection was validated (Fig. 3). Curve *a* corresponds to the blank control. Curve *b* representing an unmethylated sample only exhibits a negligible fluorescence signal, indicating that the reporter probe non-specifically adsorbed on the microbead surface barely emitted fluorescence. In contrast, the methylated sample emitted a significantly higher fluorescence signal (curve *c*), suggesting the occurrence of LDR. Therefore, methylated and unmethylated DNAs can be well differentiated through the binding of fluorescent dye-labeled reporter probe to the target sequence.

### Evaluation of sensor signal amplification

To evaluate the signal amplification of the proposed fluorescent biosensor, we compared the fluorescence signal generated by the dual signal amplification system based on dendritic DNA and polystyrene microbeads with that of microbeads only (Additional file 1: Figure S4). In the presence of 1 nM target DNA, the response signal of the dual signal amplification strategy was about (4.32 ± 0.14) times stronger than that of polystyrene microbeads alone, indicating that the proposed strategy prominently augmented the biosensor sensitivity.

### Optimization of experimental conditions

To optimize the analytical performance, we evaluated the effects of reporter probe concentration, hybridization temperature, hybridization time and ligase concentration by changing one experimental condition while maintaining others constant (Additional file 1: Figure S5). Finally, the conditions were optimized as 80 nM reporter probe, hybridization at 38 °C for 90 min and 1.5 U ligase.

### Performance of sensing system

**Fig. 4:**
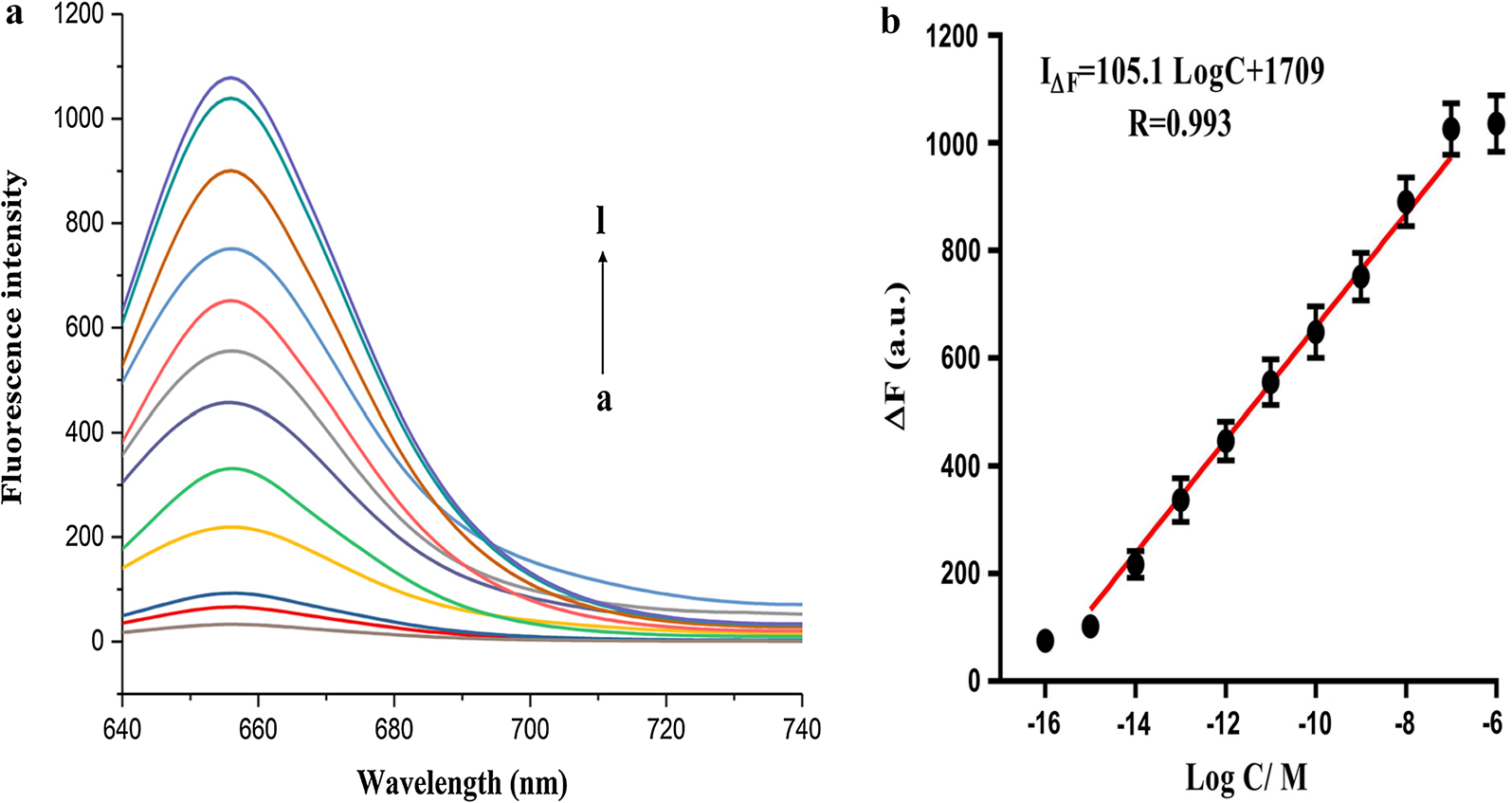
**a** Fluorescence emission spectra of methylated DNA sequences at different concentrations. a–l: 0 M, 10^−16^ M, 10^−15^ M, 10^−14^ M, 10^−13^ M, 10^−12^ M, 10^−11^ M, 10^−10^ M, 10^−9^ M, 10^−8^ M, 10^−7^ M and 10^−6^ M; **b** linear relationship between fluorescence intensity and logarithm of methylated DNA concentration

To study the sensitivity of the proposed strategy, the fluorescence intensities after addition of different concentrations of methylated target sequence of BRCA1 gene were measured under optimal conditions (Fig. 4). Clearly, with rising concentration (10^−16^–10^−7^ M) of methylated sequence, the fluorescence signal was gradually enhanced. The regression equation was I_∆F_ = 105.1 logC + 1709 (R: 0.993), where ∆F = F–F0 (F: fluorescence signal of methylated DNA; F0: blank signal), I∆F is the relative fluorescence intensity and C is the methylated DNA concentration. Notably, LOD was 0.4 fM (based on 3σ/slope) [54]. As evidenced by both low LOD and wide linear range, this ultrasensitive fluorescent biosensor can be applied to detect low-level DNA methylation. Meanwhile, this method was superior to previously reported ones (Additional file 1: Table S2).

### Sensor specificity and reproducibility

The performance of the designed sensor was further tested by studying its specificity (Additional file 1: Figure S6) and reproducibility (Additional file 1: Figure S7). The specificity was confirmed through exposure to three types of target sequences under identical conditions, including methylated, unmethylated and non-complementary DNAs. The fluorescence intensity of blank control was basically the same as those of 1 pM unmethylated and non-complementary DNAs. However, the fluorescence intensity of 1 pM methylated DNA was markedly raised, suggesting high selectivity of the proposed biosensor.

We then tested the reproducibility of the proposed method by independently detecting 1 pM methylated DNA with six freshly prepared biosensors under identical conditions. The fluorescence intensities of 5-methylcytosine measured three consecutive times had a relative standard deviation (RSD) of 2.86% (Additional file 1: Figure S7), indicating that this method was reproducible.

### Analysis of actual samples

Afterwards, we replaced reaction buffer with undiluted human serum, and repeated the above-mentioned test to assess the clinical feasibility of this strategy (Table 1). Methylated target sequences at five different concentrations were added into the reaction system. The recoveries ranged from 98.60% to 104.00%, and RSDs ranged from 3.34% to 6.06%. Taken together, the buffer background hardly interfered with this detection method.

**Table 1:** Experimental results of the recovery test in human serum (n = 3)

Sample	Spiked	Found	Recovery (%)	RSD (%)
1	1.00 fM	1.04 fM	104.00	5.66
2	5.00 fM	5.14 fM	102.84	4.38
3	50.00 fM	49.30 fM	98.60	6.06
4	500.00 fM	492.95 fM	98.95	4.19
5	5.00 pM	4.95 pM	99.00	3.34

Moreover, the stability of a biosensor is critical to its practical application, especially in complex biomedical environments. Therefore, we evaluated the effects of 28 days of storage at 4 °C, nuclease and cell lysate on the stability of the constructed fluorescent biosensor. Additional file 1: Figure S8A shows the fluorescence signals of 1 pM methylated target DNA measured for the first time and every 7 days. The fluorescence intensity gradually decreased with extended time and remained as high as 92.47% of the original one on the 28th day. The fluorescence intensity barely changed after treatment of dendritic DNA with cell lysate for 0–5 h (Additional file 1: Figure S8B) and with DNase I (1 U/mL, a considerably higher concentration than that in living cells) for 0–24 h (Additional file 1: Figure S8C). Hence, this biosensor had high stability.

**Fig. 5:**
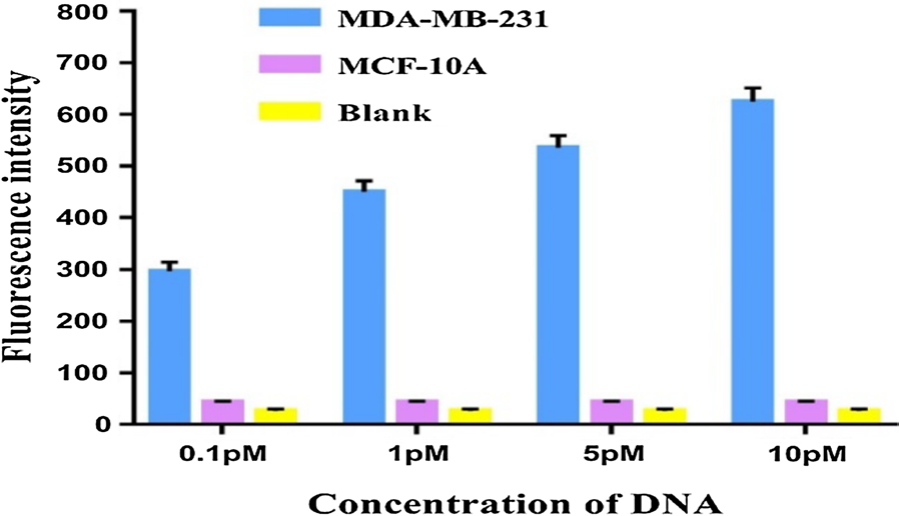
Fluorescence emission intensities of BRCA1 with methylation in the promoter region in breast cancer and normal cells

We further applied this method to study the methylation status of CpG island in the BRCA1 promoter in breast cancer MDA-MB-231 cells and actual tissue samples. According to the study of Naushad et al. [55], MDA-MB-231 cells with BRCA1 hypermethylation were herein selected as a positive experimental group, and MCF-10A cells were used as a negative control group. The fluorescence intensity of the MDA-MB-231 group increased with rising amount of DNA, whereas the feeble fluorescence intensity of the MCF-10A group remained unchanged (Fig. 5), indicating that the methylation status of breast cancer cells can be sensitively analyzed by this method.

**Fig. 6:**
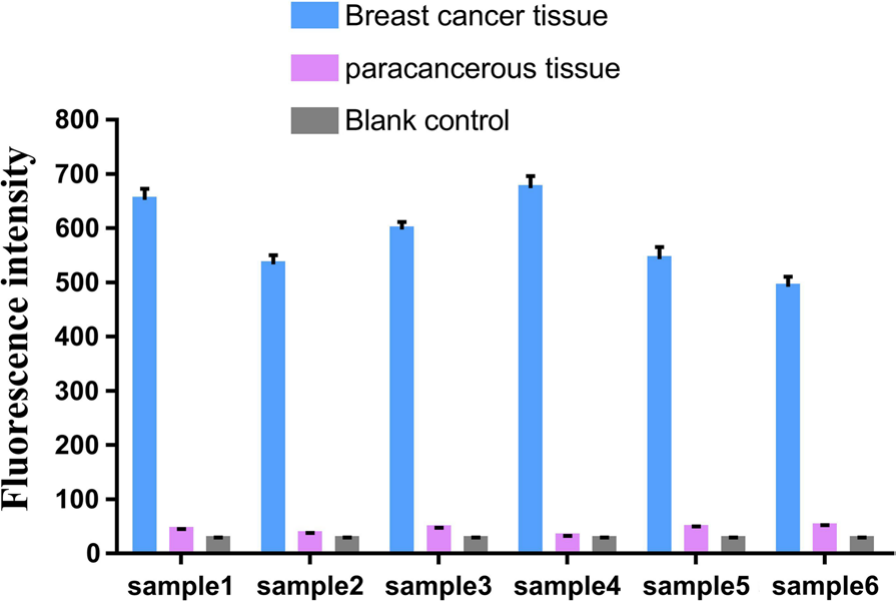
Fluorescence emission intensities of BRCA1 with methylation in the promoter region in human genomic DNA sample

The designed strategy was further validated by using human genomic DNA with methylation at − 28 bp of the BRCA1 promoter. After PCR amplification, the product exhibits a strong band between 100 and 200 bp, being consistent with the length of the target (136 bp) (Additional file 1: Figure S9). In the presence of 1 nM DNA, the fluorescence intensity of adjacent tissue was significantly lower than that of breast cancer tissue (Fig. 6). The results are in good agreement with those of sequencing (Additional file 1: Figure S10). Accordingly, the methylation status of breast cancer samples can also be sensitively analyzed by the proposed biosensor.

## Conclusions

In summary, we herein reported a simple, sensitive, rapid and novel fluorescence-based method for specifically detecting DNA methylation. Through an enzyme-free DNA-catalyzed hairpin structure based on strand displacement, the signal of dendritic DNA was subjected to cascade amplification, thereby allowing ultrasensitive, label-free detection of DNA methylation. This method had an LOD of 0.4 fM which was much lower than those of most currently available methods. In combination with LDR, this method also had high specificity. In addition, the method was facile and fast, without needing complicated operation steps such as PCR amplification. Notably, DNA methylation detection can be completed within only 2 h. Hence, this strategy is potentially applicable to the early diagnosis of breast cancer and other related diseases.

## Material and methods

### Reagents and apparatus

All oligonucleotide sequences were synthesized by Sangon Biotech (Shanghai) Co., Ltd. (China) (Additional file 1: Table S1). All oligonucleotide stock solutions were prepared by using TE buffer and stored at −20 °C. 3-Aminopropyltriethoxysilane was purchased from Sigma-Aldrich (USA). Streptavidin-coated microspheres (size: 5.5 μm) were bought from Bangs Laboratories (USA). Taq DNA ligase was obtained from NEB (USA). EZ DNA Methylation-Gold^TM^ kit was provided by Zymoresearch (USA). DNA Mini genomic DNA extraction kit was purchased from Qiagen (USA). Deionized water with a resistivity of over 18 MΩ cm was prepared by Millipore Milli-Q system (USA). All other chemicals were of analytical grade.

Agarose gel electrophoresis was performed on Biometra gel electrophoresis apparatus (Germany). DLS was carried out to measure particle sizes, and data were analyzed with Malvern ZEN3690 particle sizer (UK). Fluorescence measurements were conducted with Hitachi F-7000 fluorescence spectrometer (Japan).

### Synthesis of dendritic DNA nanopolymer

Eight DNA hairpin structures were designed (Additional file 1: Table S1). Four kinds of hairpin probes H1, H2, H3 and H4 were denatured at 95 °C for 5 min, and cooled to room temperature at a rate of 1 °C/min. Afterwards, excitation probe P as well as hairpin probes H1, H2, H3 and H4 were dissolved in buffer (10 mM Tris, pH 8.0, 1 mM EDTA, 50 mM NaCl, 12.5 mM MgCl_2_) to the final concentrations of 5 nM, 100 nM, 100 nM, 100 nM and 100 nM, respectively. Then they were incubated at 25 °C for 60 min to synthesize X-DNA which was referred to as X-DNA.

Similarly, four hairpin DNAs G0, G1, G2 and G3 were designed. G0, G2 and G3 or G1, G2 and G3 were mixed and incubated to synthesize two types of Y-DNAs which were referred to as Y0-DNA and Y1-DNA, respectively. Y0-DNA, Y1-DNA and X-DNA were mixed at a molar ratio of 1:6:2 to form a dendritic DNA polymer which was finally reinforced by adding a non-specific cross-linking agent psoralen [56, 57]. The assembled dendritic DNA was stored at 4 °C for 60 days to test its stability.

### Characterizations of dendritic DNA

Dendritic DNA was characterized by using TEM, AFM, DLS and agarose gel electrophoresis. For agarose gel electrophoresis, 1 μL of loading buffer and 5 μL of sample were uniformly mixed, loaded into each lane, and electrophoresed at 100 V for 5 min and then at 80 V for 60 min.

### Bisulfite treatment of DNA

DNA was subjected to bisulfite conversion using EZ DNA Methylation-Gold^TM^ kit according to the manufacturer’s instructions. Briefly, 130 μL of CT conversion reagent was added into 20 μL of DNA sample, and placed in a thermocycler at 98 °C for 10 min and at 64 °C for 3.5 h. After being cooled to 4 °C, the sample was transferred to a Zymo-Spin IC column containing 600 μL of M-binding buffer, and mixed upside down. Then the column was centrifuged at 10,000×*g* for 30 s. After removal of the supernatant, the residue was added 200 μL of M-desulphonation buffer, left still for 15–20 min, and centrifuged for 30 s. Subsequently, 200 μL of M-wash buffer was added into the column, centrifuged for 30 s, added 200 μL of M-wash buffer and centrifuged for another 30 s. The column was thereafter placed in a 1.5 mL centrifuge tube, added 10 μL of M-elution buffer and centrifuged for 30 s to elute DNA. The obtained DNA was used immediately or stored at − 20 °C.

### Microbead functionalization of DNA probe

Microbeads were prepared according to a modified procedure of instructions, and bound with DNA capture probe to form a complex (MB-CP). Avidin-coated microbeads (1 μM) were washed twice with 100 μL of binding buffer (20 mM Tris, pH 7.5, 1 M NaCl, 1 mM EDTA, 0.0005% Triton X-100), and centrifuged at 10,000×*g* for 3 min. The resulting supernatant was discarded. The microbeads were resuspended in 20 μL of binding buffer and added 5–10 μg of biotinylated capture probe. The final bead concentration was kept at 40 mg/mL. The mixture was gently shaken for 15 min at room temperature with a vortex mixer and centrifuged. After the supernatant was discarded, the residue was washed twice again with 100 μL of binding buffer to remove the unbound oligonucleotides. After centrifugation, the precipitate was resuspended in 100 μL of binding buffer and stored at 4 °C prior to use.

### Sample detection

In 20 μL of hybridization buffer, 20 μL of MB-CP and target sequence were well mixed, incubated for 1 h in dark at the optimum temperature, added 20 μL of hybridized conjugate of dendritic DNA/reporter probe, and hybridized under mild stirring for 1 h in dark at the optimum temperature. After centrifugation to discard the supernatant, the residue was washed 3 times with 100 μL of hybridization buffer to remove excess unbound conjugate. DNA ligase was added to the microbead conjugate suspension to trigger LDR at 94 °C for 30 s and at 56 °C for 3 min. The product was then centrifuged, and the residue was washed twice with 0.1 M pre-warmed PBS (pH 7.0, containing 0.2% Tween 20) and doubly distilled water. Methylated and unmethylated LDR products were referred to as MMB and UMMB, respectively, and dissolved in 100 μL of hybridization buffer each, from which 3 μL was characterized by SEM. Afterwards, each LDR product was dissolved in 1 mL of hybridization buffer that was transferred to clean quartz cells for fluorescence spectroscopy using Hitachi F-7000 fluorescence spectrometer controlled by FL Solution software. The emission wavelength range was 500–800 nm, the excitation wavelength was 625 nm, and the excitation and emission slits were both 10 nm. The performance of the proposed sensing system was assessed using the fluorescence intensity at 650 nm. All measurements were carried out at room temperature.

### Collection of clinical samples and preparation of genomic DNA

MCF10A cells were cultured in DMEM/F12 medium containing 5% horse serum, 20 ng/ml epithelial growth factor, 0.5 mg/ml hydrocortisone, 100 ng/ml cholera toxin, 10 μg/ml insulin, 100 IU/mL penicillin and 100 μg/mL streptomycin. MDA-MB-231 cells were cultured in DMEM supplemented with 10% fetal bovine serum, 100 IU/mL penicillin and 100 μg/mL streptomycin. All cells were cultured in a 37 °C incubator with 5% CO_2_, and those in the logarithmic growth phase were prepared into a suspension. Genomic DNA was extracted using TIANamp genomic DNA kit (Tiangen Biotech (Beijing) Co., Ltd., China). Fresh normal breast tissue and breast cancer tissue samples were collected from Department of Breast Surgery, Affiliated Hospital of Guizhou Medical University. All tissue samples were in accordance with the WHO criteria and histologically examined by senior pathologists in Department of Pathology of our hospital. In the early morning, 8 mL of fasting peripheral blood was taken from patients with breast cancer, naturally coagulated at room temperature and centrifuged at 4000 rpm for 10 min. The resulting serum was stored at − 80 °C prior to use. This study has been approved by the ethics committee of our hospital and conducted in accordance with ethical guidelines. Genomic DNA of tissue samples was extracted by TIANamp FFPE DNA kit (Tiangen Biotech (Beijing) Co., Ltd., China).

### PCR amplification and methylation sequencing

After bisulfite conversion of the extracted genomic DNA, a 50 μL reaction system was used for PCR: 1.25 U Hot Master *Taq* polymerase, 5 μL of 10 × Taq buffer, 0.1 μM forward and reverse primers (forward primer: ATGTGTTTGAGGAGGATTTA; reverse primer: ATTTCTAATTATTTTCTTTTTCTTACA), 40 μM dNTP and 20 ng genomic DNA. There were 30 cycles of reaction at 95 °C for 30 s, at 58 °C for 30 s and at 65 °C for 30 s, as well as 5 min of extension at 65 °C. The methylation status was examined by DNA methylation sequencing as described above.

## Supplementary information

**Additional file 1.** Additional tables and figures.

## Data Availability

All data generated and analyzed during this study are included in this published article.

## Abbreviations

AFM: atomic force microscopy
DLS: dynamic light scattering
LDR: ligation detection reaction
LOD: limit of detection
RSD: relative standard deviation
SEM: scanning electron microscopy
TdT: terminal deoxynucleotidyl transferase
TEM: transmission electron microscopy

## References

[1] Jiang Y, Liu S, Chen X, Cao Y, Tao Y (2013). Genome-wide distribution of DNA methylation and DNA demethylation and related chromatin regulators in cancer. Biochim Biophys Acta.

[2] He XJ, Chen T, Zhu JK (2011). Regulation and function of DNA methylation in plants and animals. Cell Res.

[3] Jones PA (2012). Functions of DNA methylation: islands, start sites, gene bodies and beyond. Nat Rev Genet.

[4] Schübeler D (2015). Function and information content of DNA methylation. Nature.

[5] Gkountela S, Castro GF, Szczerba BM, Vette M, Landin J, Ramona S (2019). Circulating tumor cell clustering shapes DNA methylation to enable metastasis seeding. Cell.

[6] Klutstein M, Nejman D, Greenfield R, Cedar H (2016). DNA methylation in cancer and aging. Cancer Res.

[7] Sina AAI, Carrascosa LG, Liang ZY, Grewal YS, Wardiana A, Shiddiky MJA (2018). Epigenetically reprogrammed methylation landscape drives the DNA self-assembly and serves as a universal cancer biomarker. Nat Commun.

[8] Smith ZD, Meissner A (2013). DNA methylation: roles in mammalian development. Nat Rev Genet.

[9] Moore LD, Le T, Fan G (2013). DNA methylation and its basic function. Neuropsychopharmacology.

[10] Boyne DJ, O’Sullivan DE, Olij BF, King WD, Friedenreich CM, Brenner DR (2018). Physical activity, global DNA methylation and breast cancer risk: a systematic literature review and meta-analysis. Cancer Epidemiol Biomarkers Prev.

[11] Polak P, Kim J, Braunstein LZ, Karlic R, Haradhavala NJ, Tiao G (2017). A mutational signature reveals alterations underlying deficient homologous recombination repair in breast cancer. Nat Genet.

[12] Evans DGR, van Veen EM, Byers HJ, Wallace AJ, Ellingford JM, Beaman G (2018). A dominantly inherited 5′ UTR variant causing methylation-associated silencing of BRCA1 as a cause of breast and ovarian cancer. Am J Hum Genet.

[13] Anjum S, Fourkala EO, Zikan M, Wong A, Gentry-Maharaj A, Jones A (2014). A BRCA1-mutation associated DNA methylation signature in blood cells predicts sporadic breast cancer incidence and survival. Genome Med.

[14] Cheow LF, Quake SR, Burkholder WF, Messerschmidt DM (2015). Multiplexed locus-specific analysis of DNA methylation in single cells. Nat Protoc.

[15] Rand KN, Young GP, Ho T, Molloy PL (2013). Sensitive and selective amplification of methylated DNA sequences using helper-dependent chain reaction in combination with a methylation-dependent restriction enzymes. Nucleic Acids Res.

[16] Stevens M, Cheng JB, Li D, Xie M, Hong C, Maire CL (2013). Estimating absolute methylation levels at single-CpG resolution from methylation enrichment and restriction enzyme sequencing methods. Genome Res.

[17] Yegnasubramanian S, Lin X, Haffner MC, DeMarzo AM, Nelson WG (2006). Combination of methylated-DNA precipitation and methylation-sensitive restriction enzymes (COMPARE-MS) for the rapid, sensitive and quantitative detection of DNA methylation. Nucleic Acids Res.

[18] Krueger F, Andrews SR (2011). Bismark: a flexible aligner and methylation caller for Bisulfite-Seq applications. Bioinformatics.

[19] Yuan XL, Gao N, Xing Y, Zhang HB, Zhang AL, Liu J (2016). Profiling the genome-wide DNA methylation pattern of porcine ovaries using reduced representation bisulfite sequencing. Sci Rep..

[20] Lee EJ, Luo J, Wilson JM, Shi H (2013). Analyzing the cancer methylome through targeted bisulfite sequencing. Cancer Lett.

[21] Adusumalli S, Mohd Omar MF, Soong R, Benoukraf T (2015). Methodological aspects of whole-genome bisulfite sequencing analysis. Brief Bioinform.

[22] Mulqueen RM, Pokholok D, Norberg SJ, Torkenczy KA, Fields AJ, Sun D (2018). Highly scalable generation of DNA methylation profiles in single cells. Nat Biotechnol.

[23] Kitchen MO, Bryan RT, Emes RD, Luscombe CJ, Cheng KK, Zeegers MP (2018). Humanmethylation450K array-identified biomarkers predict tumour recurrence/progression at initial diagnosis of high-risk non-muscle invasive bladder cancer. Biomark Cancer.

[24] Bibikova M, Barnes B, Tsan C, Ho V, Klotzle B, Le JM (2011). High density DNA methylation array with single CpG site resolution. Genomics.

[25] Wilhelm-Benartzi CS, Koestler DC, Karagas MR, Flanagan JM, Christensen BC, Kelsey KT (2013). Review of processing and analysis methods for DNA methylation array data. Br J Cancer.

[26] Yang R, Pfütze K, Zucknick M, Sutter C, Wappenschmidt B, Marme F (2015). DNA methylation array analyses identified breast cancer-associated HYAL2 methylation in peripheral blood. Int J Cancer.

[27] Wang LJ, Han X, Li CC, Zhang CY (2018). Single-ribonucleotide repair-mediated ligation-dependent cycling signal amplification for sensitive and specific detection of DNA methyltransferas. Chem Sci.

[28] Cui L, Hu J, Wang M, Li CC, Zhang CY (2019). Label-free and immobilization-free electrochemical Magnetobiosensor for sensitive detection of 5-hydroxymethylcytosine in genomic DNA. Anal Chem.

[29] Huang J, Zhang S, Mo F, Su S, Chen X, Li Y (2019). An electrochemical DNA biosensor analytic technique for identifying DNA methylation specific sites and quantify DNA methylation level. Biosens Bioelectron.

[30] Wu Q, Amrutkar SM, Shao F (2018). Sulfinate based selective labelling of 5-hydroxy methylcytosine: application to biotin pull down assay. Bioconjug Chem.

[31] Yotani T, Yamada Y, Arai E, Tian Y, Gotoh M, Komiyama M (2018). Novel method for DNA methylation analysis using high-performance liquid chromatography and its clinical application. Cancer Sci.

[32] Poh WJ, Wee CP, Gao Z (2016). DNA Methyltransferase activity assays: advances and challenges. Theranostics.

[33] Yang Q, Li J, Wang X, Peng H, Xiong H, Chen L (2018). Strategies of molecular imprinting-based fluorescence sensors for chemical and biological analysis. Biosens Bioelectron.

[34] Chen S, Ma H, Li W, Nie Z, Yao S (2017). An entropy-driven signal amplifying strategy for real-time monitoring of DNA methylation process and high-throughput screening of methyltransferase inhibitors. Anal Chim Acta.

[35] Zhao Y, Chen F, Wu Y, Dong Y, Fan C (2013). Highly sensitive fluorescence assay of DNA methyltransferase activity via methylation-sensitive cleavage coupled with nicking enzyme-assisted signal amplification. Biosens Bioelectron.

[36] Feng F, Liu L, Wang S (2010). Fluorescent conjugated polymer-based FRET technique for detection of DNA methylation of cancer cells. Nat Protoc.

[37] Jiang B, Wei Y, Xu J, Yuan R, Xiang Y (2017). Coupling hybridization chain reaction with DNAzyme recycling for enzyme-free and dual amplified sensitive fluorescent detection of methyltransferase activity. Anal Chim Acta.

[38] Xue Q, Lv Y, Xu S, Zhang Y, Wang L, Li R (2015). Highly sensitive fluorescence assay of DNA methyltransferase activity by methylation-sensitive cleavage-based primer generation exponential isothermal amplification-induced G-quadruplex formation. Biosens Bioelectron.

[39] Zhou X, Zhao M, Duan X, Guo B, Cheng W, Ding S (2017). Collapse of DNA tetrahedron nanostructure for “Off-On” fluorescence detection of DNA methyltransferase activity. ACS Appl Mater Interfaces.

[40] Wang ZY, Wang LJ, Zhang QY, Tang B, Zhang CY (2018). Single quantum dot-based nanosensor for sensitive detection of 5-methylcytosine at both CpG and non-CpG sites. Chem Sci.

[41] Wang ZY, Wang M, Zhang Y, Zhang CY (2018). Sensitive and label-free discrimination of 5-hydroxymethylcytosine and 5-methylcytosine in DNA by ligation-mediated rolling circle amplification. Chem Commun (Camb).

[42] Zhang Y, Wang XY, Zhang Q, Zhang CY (2017). Label-free sensitive detection of DNA methyltransferase by target-induced hyperbranched amplification with zero background signal. Anal Chem.

[43] Hori Y, Otomura N, Nishida A, Nishiura M, Umeno M, Suetake I (2018). Synthetic-molecule/protein hybrid probe with fluorogenic switch for live-cell imaging of DNA methylation. J Am Chem Soc.

[44] He H, Dai J, Duan Z, Meng Y, Zhou C, Long Y (2016). Target-catalyzed autonomous assembly of dendrimer-like DNA nanostructures for enzyme-free and signal amplified colorimetric nucleic acids detection. Biosens Bioelectron.

[45] Li Y, Cu YT, Luo D (2005). Multiplexed detection of pathogen DNA with DNA-based fluorescence nanobarcodes. Nat Biotechnol.

[46] Zhou T, Chen P, Niu L, Jin J, Liang D, Li Z (2012). pH-responsive size-tunable self-assembled DNA dendrimers. Angew Chem Int Ed Engl.

[47] Um SH, Lee JB, Kwon SY, Li Y, Luo D (2006). Dendrimer-like DNA-based fluorescence nanobarcodes. Nat Protoc.

[48] Lei C, Huang Y, Nie Z, Hu J, Li L, Lu G (2014). A supercharged fluorescent protein as a versatile probe for homogeneous DNA detection and methylation analysis. Angew Chem Int Ed Engl.

[49] Böhme K, Cremonesi P, Severgnini M, Villa TG, Fernández-No IC, Barros-Velázquez J (2014). Detection of food spoilage and pathogenic bacteria based on ligation detection reaction coupled to flow-through hybridization on membranes. Biomed Res Int.

[50] Cremonesi P, Pisoni G, Severgnini M, Consolandi C, Moroni P, Raschetti M (2009). Pathogen detection in milk samples by ligation detection reaction-mediated universal array method. J Dairy Sci.

[51] Liu H, Luo J, Fang L, Huang H, Deng J, Huang J (2018). An electrochemical strategy with tetrahedron rolling circle amplification for ultrasensitive detection of DNA methylation. Biosens Bioelectron.

[52] Meng HM, Zhang X, Lv Y, Zhao Z, Wang NN, Fu T (2014). DNA dendrimer: an efficient nanocarrier of functional nucleic acids for intracellular molecular sensing. ACS Nano.

[53] Li L, Niu C, Li T, Wan Y, Zhou Y, Wang H (2018). Ultrasensitive electrochemiluminescence biosensor for detection of laminin based on DNA dendrimer-carried luminophore and DNA nanomachine-mediated target recycling amplification. Biosens Bioelectron.

[54] Kong RM, Song ZL, Meng HM, Zhang XB, Shen GL, Yu RQ (2014). A label-free electrochemical biosensor for highly sensitive and selective detection of DNA via a dual-amplified strategy. Biosens Bioelectron.

[55] Naushad SM, Reddy CA, Kumaraswami K, Divyya S, Kotamraju S, Gottumukkala SR (2014). Impact of hyperhomocysteinemia on breast cancer initiation and progression: epigenetic perspective. Cell Biochem Biophys.

[56] Wang J, Jiang M, Nilsen TW, Getts RC (1998). Dendritic nucleic acid probes for DNA biosensors. J Am Chem Soc.

[57] Nilsen TW, Grayzel J, Prensky W (1997). Dendritic nucleic acid structures. J Theor Biol.

