# Supplementary material for: A novel fluorescent biosensor based on dendritic DNA nanostructure in combination with ligase reaction for ultrasensitive detection of DNA methylation
Source: J Nanobiotechnology. 2019 Dec 7;17:121. doi: 10.1186/s12951-019-0552-5 (PMC6898925; doi:10.1186/s12951-019-0552-5)
Supplement: Supplementary file 1 — Additional file 1. Additional tables and figures. [file 12951_2019_552_MOESM1_ESM.doc]

**A novel fluorescent biosensor based on dendritic DNA nanostructure in combination with ligase reaction for ultrasensitive detection of DNA methylation**

Shu Zhang^1,2,3^†, Jian Huang^3^†, Jingrun Lu^3^, Min Liu^3^, Yan Li^1^, Lichao Fang^1^, Hui Huang^1^, Jianjun Huang^4^, Fei Mo,^2,3^*, Junsong Zheng^1^*

1. Department of Clinical and Military Laboratory Medicine, College of Medical Laboratory Science, Army Medical University, Chongqing 400038, China

2. Department of Basic Clinical Laboratory Medicine, School of Clinical Laboratory Science, Guizhou Medical University, Guiyang 550004, China

3. Center for Clinical Laboratories, Affiliated Hospital of Guizhou Medical University, Guiyang 550004, China

4. Department of Breast Surgery, Affiliated Hospital of Guizhou Medical University, Guiyang 550004, China

†The two authors contributed equally to this study.

*Co-corresponding authors:

Junsong Zheng

E-mail: zhengalpha@tmmu.edu.cn, Tel: +86-23-68771651, Fax: +86-23-68771651

Fei Mo

Email: mofei@ gmc.edu.cn, Tel: +86-13985563839

Table S1**.** DNA sequences ^a^

| **Name** | **DNA sequence (5’ to 3’)** |
| --- | --- |
| Target DNA 1 | CCA GAC AAG GTG AAC GTG GAT GAA GTT GGT GGT GAG GTA CTA AAA ATA CA |
| Target DNA 2 | CCA GAC AAG GTG AAmC GTG GAT GAA GTT GGT GGT GAG GTA CTA AAA ATA CA |
| Mismatched DNA | TCA GGC CTG CAT GTG CTA CGT GAT GGA ATT CAG TGT CGA CGA ATA ACA AA |
| Capture probe | TAT ATT TTT AAT ACC TCA CCA CCA ACT TCA TCC AC |
| Report probe | GTT CAC CTT ATC TAA TGG ATC CGC TAG ACA TTC |
| G0 | GCT TGA GAT GTT AGG GAG TAG TGC AAC ATC CAC AAC GCA CTA CTC CCT GAA TGT CTA GCG GAT CCA |
| G1 | GCT TGA GAT GTT AGG GAG TAG TGC AAC ATC CAC AAC GCA CTA CTC CCT GCA CTA AAT GAA TGG GTT |
| G2 | AGG GAG TAG TGC GTT GTG GAT GTT AAC ATC TCA AGC AAC ATC CAC AAC CTC A |
| G3 | Cy5-GTT GTG GAT GTT GCT TGA GAT GTT GCA CTA CTC CCT AAC ATC TCA AGC AGT ACA TCA TCC ATC CTA |
| H1 | GCT TGA GAT GTT AGG GAG TAG TGC AAC ATC CAC AAC GCA CTA CTC CCT AAC CCA TTC ATT TAG TGC |
| H2 | AGG GAG TAG TGC GTT GTG GAT GTT AAC ATC TCA AGC AAC ATC CAC AAC TAG GAT GGA TGA TGT ACT |
| H3 | GTT GTG GAT GTT GCT TGA GAT GTT TTG GTC TAG GCA TGG ATG AGC CAAAAC CCA TTC ATT TAG TGC |
| H4 | TTG GCT CAT CCA TGC CTA GAC CAA GCA CTA CTC CCT AAC ATC TCA AGC TAG GAT GGA TGA TGT ACT |
| P1 | GCACTACTCCCTAACATCTCA AGC |

^a^ Red underlined sequences in G1, H1 and H3, green underlined sequences in G3, H2 and H4, and purple underlined sequences in reporter probe and G0 are designed to be complementary.


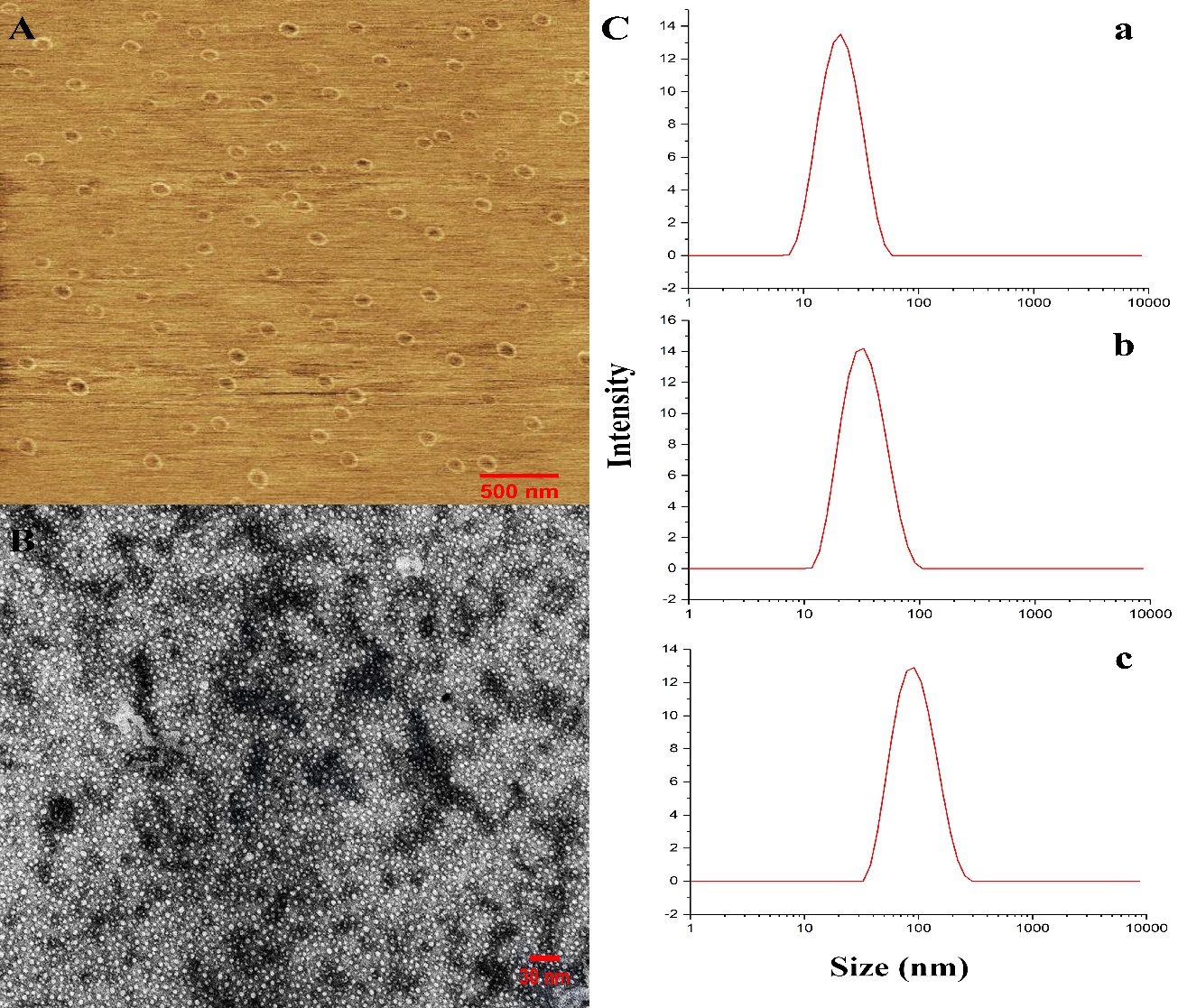


**Figure S1.** Characterizations of dendritic DNA. A: AFM image (scale bar: 500 nm); B: TEM image (scale bar: 30 nm); C: DLS results. a: X-DNA, b: Y-DNA, c: dendritic DNA.

AFM image shows that dendritic DNA has a size of (91.2 ± 6.7) nm. Due to DNA aggregation and tight intertwining induced by the self-assembly of dendritic DNA, the morphology was atypically spherical-shaped, similar to that reported by Lee et al. ^1-3^. To further study the size and distribution of dendritic DNA, TEM was performed, showing that the molecular structure of assembled dendritic DNA had high dispersibility, and the diameter was approximately 91.2 nm which was in agreement with that measured by AFM. DLS exhibited that the average particle diameters of X-DNA, Y-DNA and dendritic DNA were (23.7 ± 2.6) nm (a), (26.7 ± 7.1) nm (b) and (98.2 ± 4.8) nm (c), respectively.


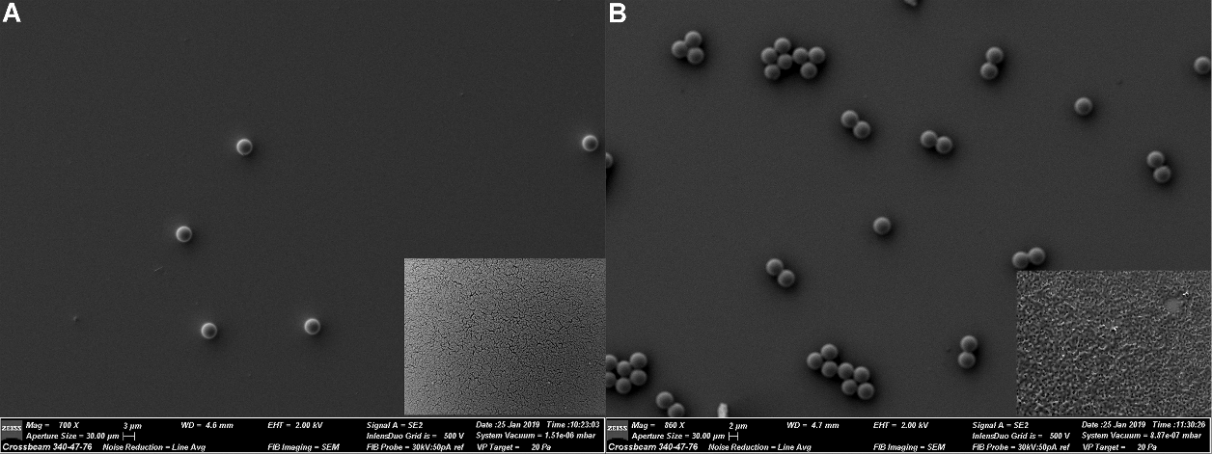


**Figure S2.** SEM images of microbead nanomaterials. A: UMMB; B: MMB.

As exhibited in Figure S2, SEM exhibited that both UMMB (Figure S2A) and MMB (Figure S2B) were spherically structured with a relatively uniform particle size distribution (mean size: 5.5μm). However, MMB had a rougher surface than that of UMMB due to covering with dendritic DNA.


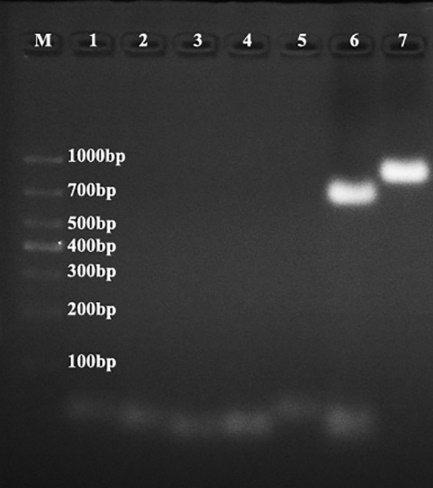


**Figure S3.** Non-denaturing gel electrophoresis results of methylated and unmethylated LDR products. Lane 1: Methylated target DNA; lane 2: unmethylated target DNA; lane 3: capture probe; lane 4: reporter probe; lane 5: ligated product of capture and reporter probes; lane 6: LDR product of unmethylated DNA target sequence; lane 7: LDR product of methylated DNA target sequence; M: DNA marker.


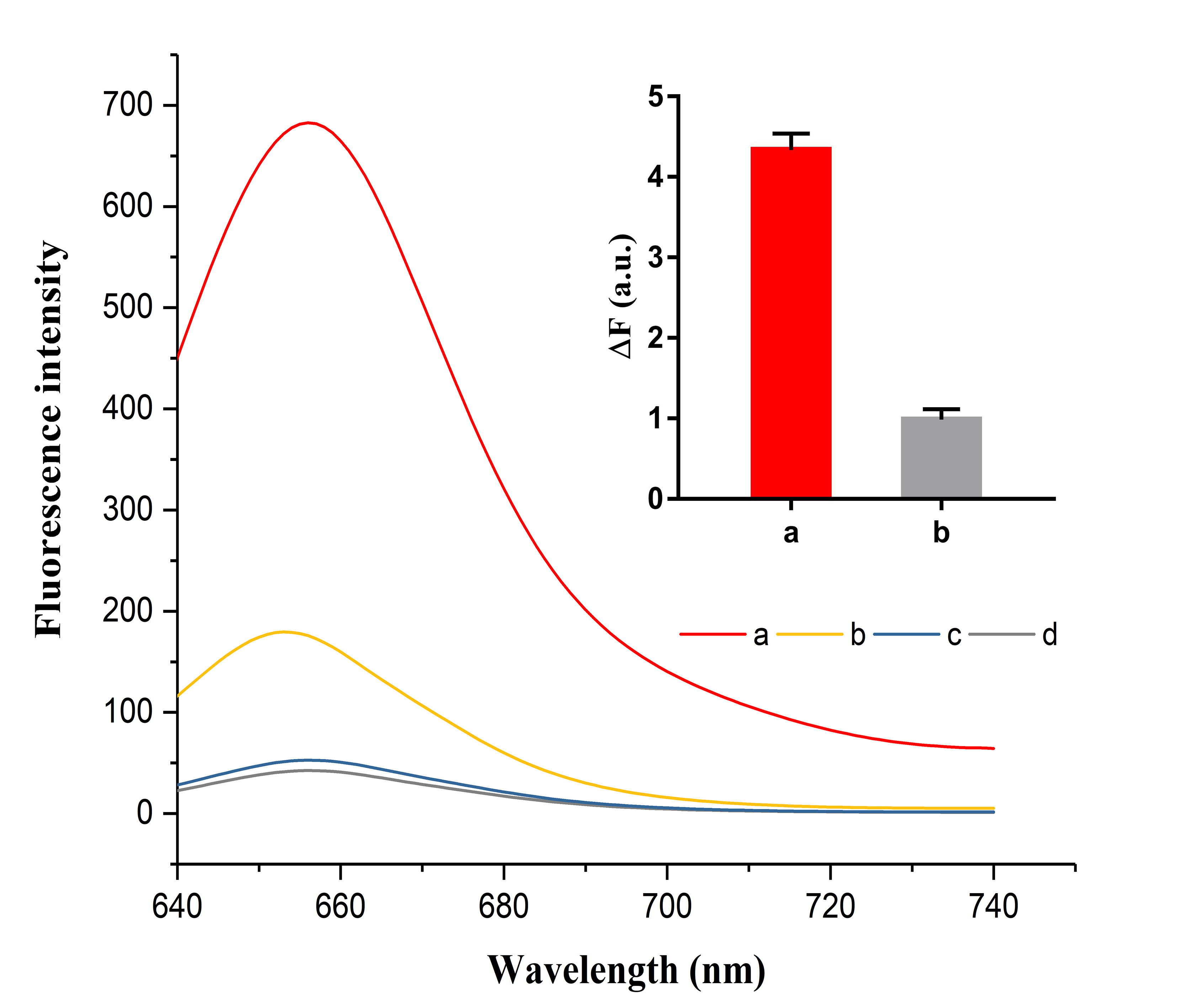


**Figure S4.** Fluorescence spectroscopy of the fluorescent biosensor under different amplification conditions: (a) fluorescence spectrum under the dual signal amplification of dendritic DNA and polystyrene microbeads with a target DNA concentration of 1 nM; (b) fluorescence spectrum in the presence of polystyrene microbeads alone with a target DNA concentration of 1 nM; (c) fluorescence spectrum under the dual signal amplification of dendritic DNA and polystyrene microbeads without target DNA; (d) fluorescence spectrum in the presence of polystyrene microbeads alone without target DNA. ΔF=(F-F0)/F0, where F and F0 represent the fluorescence signals in the presence and absence of target DNA, respectively. All experiments were repeated three times.


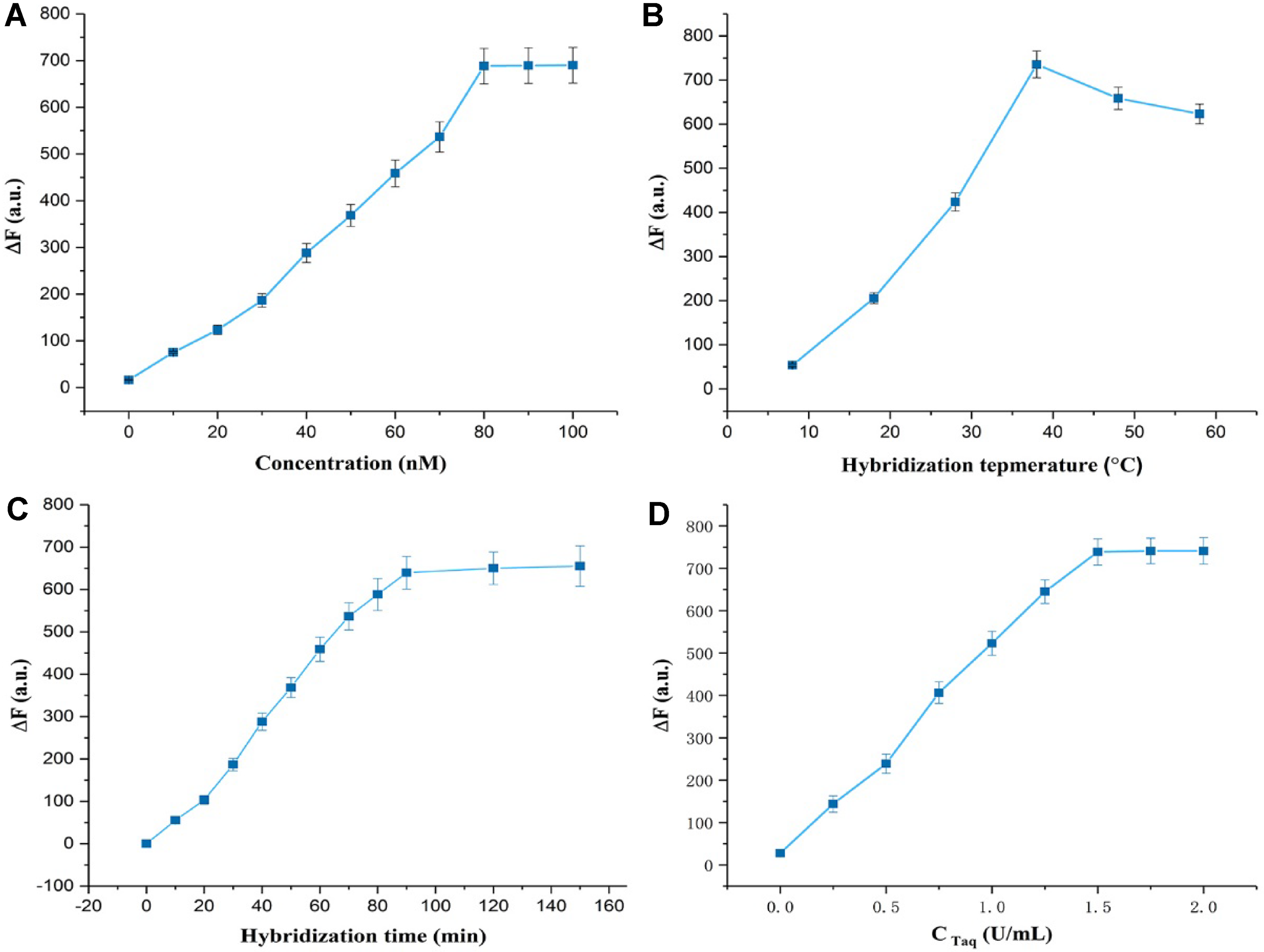


**Figure S5.** A: Effects of different concentrations (10 nM, 20 nM, 30 nM, 40 nM, 50 nM, 60 nM, 70 nM, 80 nM, 90 nM and 100 nM) of reporter probe on fluorescence detection system; B: effects of different hybridization temperatures (8°C, 18°C, 28°C, 38°C, 48°C and 58°C) on fluorescence detection system; C: effects of different hybridization times (20 min, 30 min 40 min, 50 min, 60 min, 70 min, 80 min, 90 min, 120 min, 150 min and 180 min) on fluorescence detection system; D: effects of different concentrations of ligase (0.1 U/μL, 0.25 U/μL, 0.5 U/μL, 1 U/μL, 1.25 U/μL, 1.5 U/μL, 1.75 U/μL and 2 U/μL) on fluorescence detection system. Concentration of methylated DNA: 1 nM. Standard deviation was derived from at least three independent measurements.

As displayed in Figure S5A, the reporter probe concentration affects the background signal and sensitivity of this method. On one hand, low-concentration reporter probe cannot convert all the target DNAs involved in reaction into a duplex, thus affecting the reaction efficiency. On the other hand, although reporter probe at an excessively high concentration allowed signal amplification to the maximum value, the background signal was elevated simultaneously. Accordingly, we selected a concentration range of 10~100 nM for optimization, using the concentration of reporter probe as the x axis and the relative fluorescence intensity ∆F (∆F = F - F0, F: fluorescence signal of methylated DNA; F0: blank signal) as the y axis. With rising reporter probe concentration, ∆F also increased gradually, reaching the maximum at 80 nM. As the concentration further increased, ∆F ceased rising. As a result, 80 nM was selected as the optimum probe concentration.

Hybridization temperature can affect the Gibbs free energy of nucleic acid hybridization, DNA hybridization efficiency and double strand stability. Therefore, we assessed the effects of hybridization temperature on the performance of this strategy. The influence of hybridization temperature from 8°C to 58°C on the analytical performance was then assessed (Figure S5B). At 18°C, ∆F was low, because the probe and target sequence hardly formed a stable duplex. As the hybridization temperature increased to 38°C, ∆F reached the maximum. Afterwards, ∆F gradually decreased, which can be ascribed to the adverse effects of high temperature on DNA renaturation and the enhancement of background fluorescence signal.

In the presence of sufficient substrate, hybridization time may affect DNA hybridization efficiency. Therefore, we set a total hybridization time of 2.5 h, and recorded the fluorescence intensities at 10 min, 20 min, 30 min, 60 min, 70 min, 80 min, 90 min, 120 min and 150 min. Figure S5C exhibits that ∆F soars with extended reaction time. The calibration curve shows that ∆F is proportional to the reaction time from 0 to 90 min. Hence, the reaction time was finally set at 90 min.

Moreover, the ligase concentration plays a key role in the binding efficiency of LDR ^4^. As the DNA ligase concentration increased, the fluorescence intensity rose rapidly, peaked at 1.5 U/μL and remained thereafter owing to saturation of ligase required by LDR (Figure S5D). Therefore, 1.5 U/μL ligase was selected for the following experiments.

Table S2 **Comparison with** **previous studies on DNA methylation**

| **Detection Strategy** | **Linear range/M** | **Limit of Detection (M)** | **Reference** |
| --- | --- | --- | --- |
| Differential pulse voltammetry | 1.0×10^-14^-5.0×10^-9^ | 2.0×10^-15^ | 5 |
| Colorimetric | 1.0×10^-13^-1.0×10^-9^ | 3.0×10^-14^ | 6 |
| Electrochemistry | 1.0×10^-9^-1.0×10^-13^ | 3.5×10^-14^ | 7 |
| Raman spectroscopy | 5.0×10^-12^-5.0×10^-9^ | 3.0×10^-12^ | 8 |
| Fluorescence | 1.0×10^-15^-1.0×10^-12^ | 8.0×10^-16^ | 9 |
| Fluorescence | 6.3×10^-7^-2.0×10^-9^ | 9.4×10^-10^ | 10 |
| Fluorescence | 1.0×10^-15^-1.0×10^-7^ | 4.0×10^-16^ | This work |


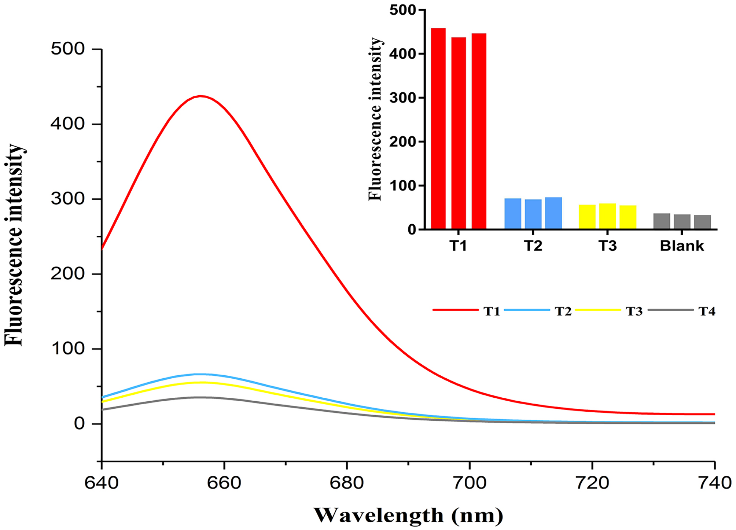


**Figure S6.** Fluorescence emission spectra of fluorescent biosensor in the presence of different targets: methylated, unmethylated and non-complementary DNAs (n=3). T1: Red, methylated DNA (concentration: 1 pM); T2: blue, unmethylated DNA (concentration: 1 pM); T3: yellow, non-complementary DNA (concentration: 1 pM); grey, blank control.


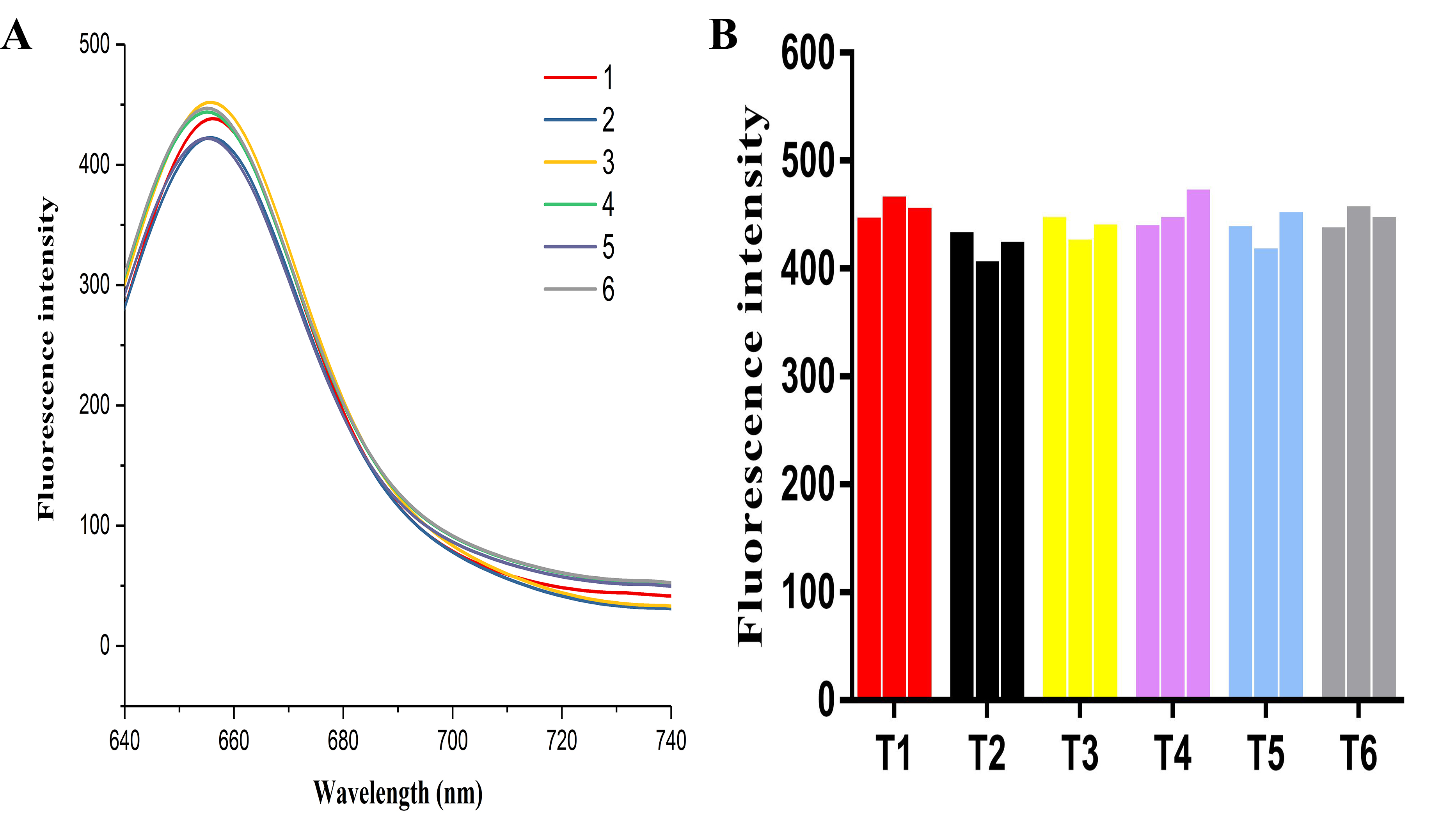


**Figure S7.** Reproducibility of proposed fluorescent biosensor. A:1-6: Fluorescence spectra of 1 pM methylated DNA detected by six freshly prepared biosensors independently; B: three repeated measurements of fluorescence intensities of 1 pM methylated DNA detected by six freshly prepared biosensors.


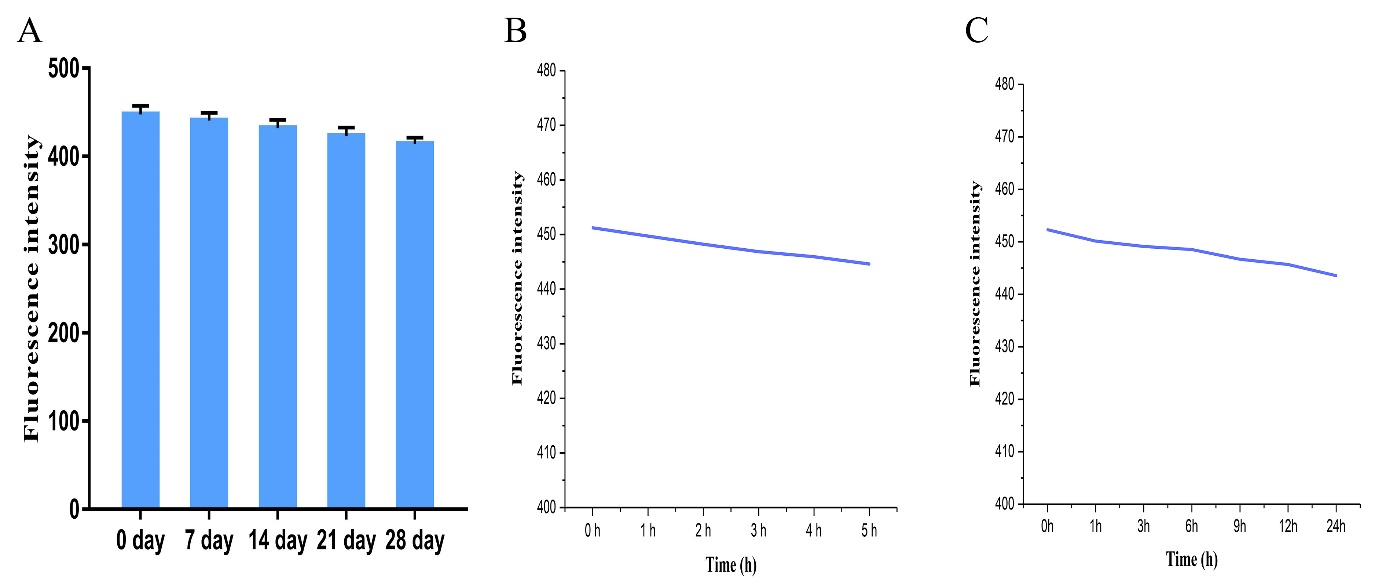


**Figure S8.** Stability of proposed fluorescent biosensor. A: Effects of storage at 4°C for 0-28 days on fluorescence intensity; B: effects of incubation of dendritic DNA with cell lysate for 0-5 h on fluorescence intensity; C: effects of incubation of dendritic DNA with DNase I for 0-24 h on fluorescence intensity. Methylated DNA concentration: 1 pM. All experiments were repeated at least three times.

**
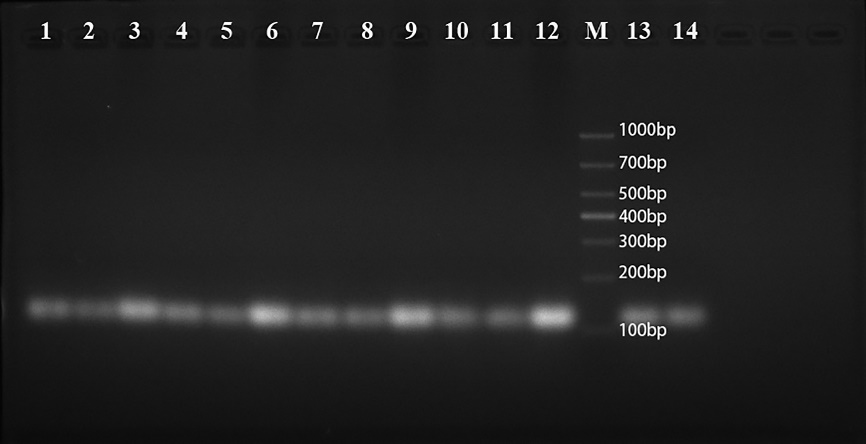
**

**Figure S9.** Agarose gel electrophoresis (2%) results of PCR products. Lanes 1-6: PCR products of breast cancer tissue; lanes 7-12: PCR products of adjacent tissue; lane 13: MDA-MB-231 cells; lane 14: MCF-10A cells; M: DNA marker.


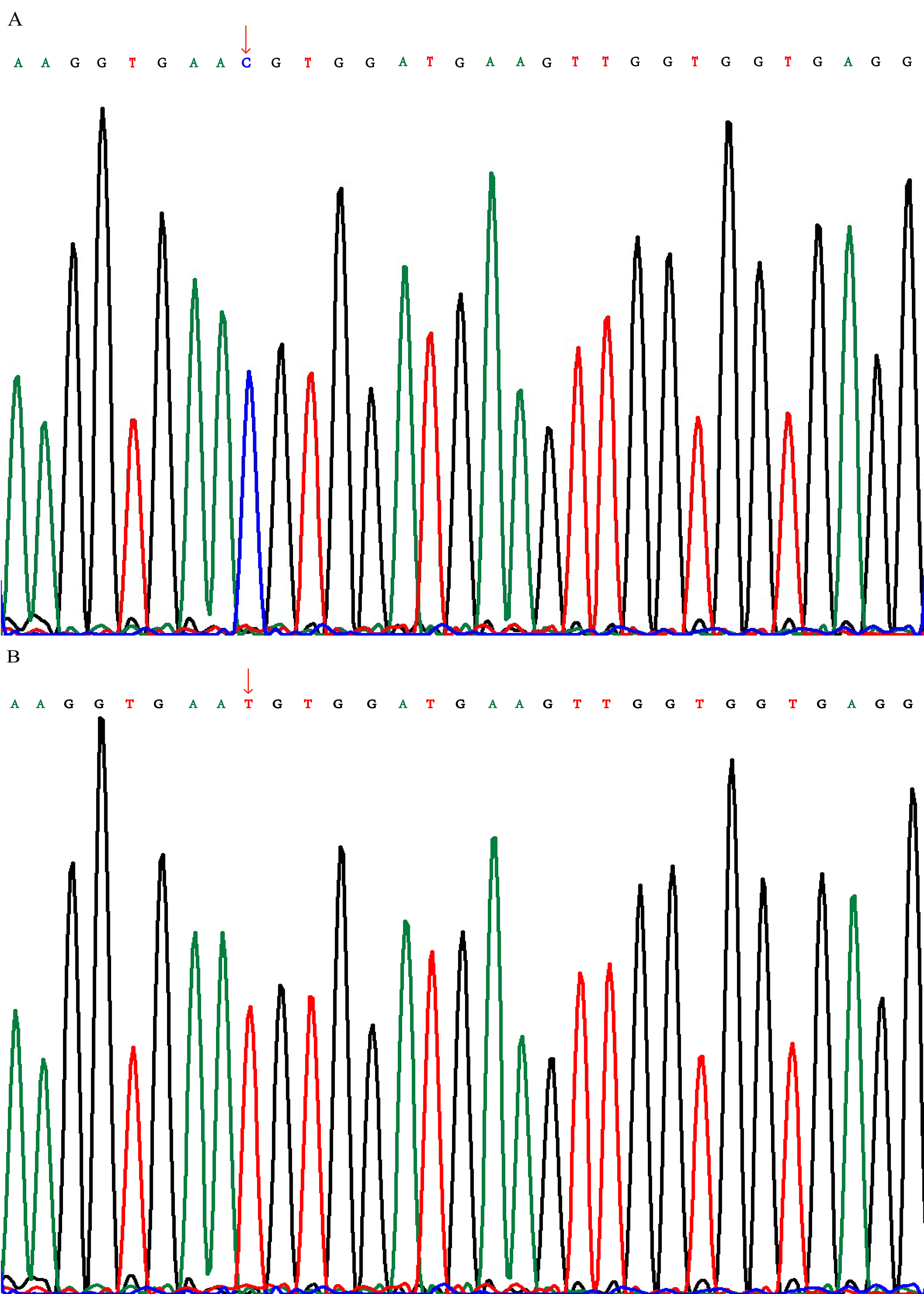


**Figure S10.** Bisulfite sequencing results of BRCA1 promoter in human genomic DNA. A: Breast cancer tissue; B: adjacent tissue. Arrow: Detection site in the promoter region of BRCA1 tumor suppressor gene.

**References**

1. Meng HM, Zhang X, Lv Y, Zhao Z, Wang NN, Fu T, et al. DNA Dendrimer: An Efficient Nanocarrier of Functional Nucleic Acids for Intracellular Molecular Sensing. ACS Nano. 2014;8:6171-81.
2. Li L, Niu C, Li T, Wan Y, Zhou Y, Wang H, et al. Ultrasensitive Electrochemiluminescence Biosensor for Detection of Laminin Based on DNA Dendrimer-Carried Luminophore and DNA Nanomachine-Mediated Target Recycling Amplification. Biosens Bioelectron. 2018;101:206-12.
3. Lee JB, Roh YH, Um SH, Funabashi H, Cheng W, Cha JJ, et al. Multifunctional nanoarchitectures from DNA-based ABC monomers. Nat Nanotechnol. 2009;4:430-6.
4. Feng K, Li J, Jiang JH, Shen GL, Yu RQ. QCM detection of DNA targets with single-base mutation based on DNA ligase reaction and biocatalyzed deposition amplification. Biosens Bioelectron. 2007;22:1651-7.
5. Daneshpour M, Syed moradi L, Izadi P, Omidfar K. Femtomolar level detection of RASSF1A tumor suppressor gene methylation by electrochemical nano-genosensor based on Fe3O4/TMC/Au nanocomposite and PT-modified electrode. Biosens Bioelectron. 2016;77:1095-103.
6. Da HM, Liu HY, Zheng YN, Yuan R, Chai YQ. A highly sensitive VEGF165 photoelectrochemical biosensor fabricated by assembly of aptamer bridged DNA networks. Biosens Bioelectron. 2018;101:213-8.
7. Yin H, Sun B, Zhou Y, Wang M, Xu Z, Fu Z, et al. A new strategy for methylated DNA detection based on photoelectrochemical immunosensor using Bi2S3 nanorods, methyl bonding domain protein and anti-his tag antibody. Biosens Bioelectron. 2014;51:103-8.
8. Hu J, Zhang CY. Single base extension reaction-based surface enhanced Raman spectroscopy for DNA methylation assay. Biosens Bioelectron. 2012;31:451- 7.
9. Cao A, Zhang CY. Sensitive and Label-Free DNA Methylation Detection by Ligation-Mediated Hyperbranched Rolling Circle Amplification. Anal Chem. 2012;84:6199-205.
10. Dadmehr M, Hosseini M, Hosseinkhani S, Reza Ganjali M, Sheikhnejad R. Label free colorimetric and fluorimetric direct detection of methylated DNA based on silver nanoclusters for cancer early diagnosis. Biosens Bioelectron. 2015;73:108-13.
